# Plant PHR Transcription Factors: Put on A Map

**DOI:** 10.3390/genes10121018

**Published:** 2019-12-06

**Authors:** Paweł Sega, Andrzej Pacak

**Affiliations:** Department of Gene Expression, Institute of Molecular Biology and Biotechnology, Faculty of Biology, Adam Mickiewicz University, Poznań, Uniwersytetu Poznańskiego 6, 61-614 Poznań, Poland; p.sega@amu.edu.pl

**Keywords:** PHR1, phosphate signaling, protein–protein interactions, post-translational modifications

## Abstract

The phosphate starvation response (PHR) protein family exhibits the MYB and coiled-coil domains. In plants, within the either 5′ untranslated regions (UTRs) or promoter regions of phosphate starvation-induced (PSI) genes are characteristic *cis*-regulatory elements, namely PHR1 binding sequence (P1BS). The most widely studied PHR protein family members, such as AtPHR1 in *Arabidopsis thaliana* (L.) and OsPHR2 in *Oryza sativa* (L.), may activate the gene expression of a broad range of *PSI* genes by binding to such elements in a phosphate (Pi) dependent manner. In Pi signaling, PHR transcription factors (TFs) can be selectively activated or deactivated by other proteins to execute the final step of signal transduction. Several new proteins have been associated with the AtPHR1/OsPHR2 signaling cascade in the last few years. While the PHR TF transcriptional role has been studied intensively, here we highlight the recent findings of upstream molecular components and other signaling pathways that may interfere with the PHR final mode of action in plants. Detailed information about transcriptional regulation of the *AtPHR1* gene itself and its upstream molecular events has been reviewed.

## 1. Introduction

Phosphorus (P) is an essential element for all living organisms. Plants acquire P as inorganic phosphate (Pi) ions. An insufficient P level in the soil is one of the most limiting factors determining crop yield and productivity. Pi rock has been mined since the late 19th century and has been used as a main source of phosphate fertilizers worldwide [1,2]. Food production experiences the effects of climate change in the form of erosion patterns that influence the pollution of surface waters, with P causing eutrophication. P is not easily available in nature because of its immobility and high reactivity with soil constituents. Thus, environmental and industrial impacts on P recycling prompt the development of balanced food production and sustainable P consumption. However, without understanding the ways in which P metabolism is regulated in eukaryotic cells, such technological efforts may not be used effectively.

The maintenance of P homeostasis in plants is strictly controlled by a molecular network regulated by a group of transcription factors (TFs). Generally, P-starved plants turn on local and long-distance signals to absorb and utilize P from either internal or external pools. Thus, the inability of live organisms to adequately adapt to P limitation allows the possibility to screen genotypes or isolate mutants for functional genomic studies. The story of P homeostasis regulators began two decades ago with the characterization of green alga *Chlamydomonas reinhardtii* (Dangeard) phosphorus-starvation response 1 (PSR1) mutants exhibiting abnormality in their response to P deprivation. Shimogawara et al. identified two mutants, *psr1-1* and *psr1-2*, that were defective in the synthesis of extracellular phosphatases and were unable to increase the rate of inorganic phosphate ion transport upon Pi scarcity [3]. They demonstrated that both mutants possess alterations in the same gene, named *PSR1*, and such variations are recessive and allelic [3]. Later, the *PSR1* gene product was further investigated and has been recognized as a central transcriptional regulator that is needed to activate specific responses to P limitation [4,5]. Subsequent studies with higher plants revealed homologous genes, phosphate starvation response 1 (*PHR1*) in *Arabidopsis thaliana* (L.) [6], and phosphate starvation response 2 *(PHR2*) in *Oryza sativa* (L.) [7], which is orthologue of the *AtPHR1* gene. Overexpression of *AtPHR1* leads to increased Pi level in the shoot tissues, together with induction of several Pi starvation-induced (PSI) genes that encode phosphate transporters, phosphatases, or RNases [8,9]. While knockout of the *AtPHR1* gene leads to defective accumulation of anthocyanin, starch, and sugar, alteration in the root architecture and impaired induction of multiple genes are known responses to Pi scarcity [10,11]. While the transcriptional regulation of many PSI genes by PHR1 TF is clear, the mechanism regulating the *PHR1* transcript level and protein activity itself still remains largely unexplored.

Many components of the complex molecular networks are still missing. Thus, here we would like to highlight the most important findings on the PHR-like protein family and PSI gene expression regulation that may determine low-Pi tolerance in crop plants.

## 2. PHRs Redundancy and Dimerization

Among the eukaryotes, high functional redundancy of transcription factors is a phenomenon that is known to lead to one TF compensating for another, masking the TF knockout effect on the binding targets [12,13]. PHR-like proteins belong to the MYB–coiled-coil (MYB-CC) family of transcription factors, which are encoded by 15 genes in *Arabidopsis*, and as dimers bind an imperfect palindromic sequence (PHR1 binding sequence (P1BS); GnATATnC) [6,14,15]. Characteristic P1BS *cis*-regulatory motifs may be found either in the promoter or 5′ untranslated regions (UTRs) of the target genes, where PHR1 TF binds acting as an activator or repressor of transcription [16,17]. Apart from AtPHR1, other MYB-CC family members were found in recent studies in *Arabidopsis*: PHL1 (PHR1-like 1) [11], PHL2 and PHL3 [18], as well as PHL4 [19]. First remarks about PHR1 functional redundancy were found in *phr1 phl1* double mutant in *Arabidopsis*. The loss-of-function double mutation only partially affected the transcription of *PSI* genes indicating the synergistic effect of *PHR1/PHL1* genes and involvement of other PHR-like TFs [11].

### 2.1. Cooperation between PHR Family Members

In particular, studies in various plant species demonstrated the widespread species-specific functions of PHR-like TFs. Overexpression of *TaPHR1* resulted in upregulation of a subset of PSI genes following the stimulation of lateral root branching and overall grain yield promotion of *Triticum aestivum* (L.) plants under Pi scarcity [20]. On the contrary, overexpression of *BnPHR1* caused Pi accumulation in shoots and retarded growth of *Brassica napus* (L.) plants [21]. Relevant work in rice has disclosed a few more *AtPHR1* orthologues, such as *OsPHR1*, *OsPHR2*, *OsPHR3* [7,22], and *OsPHR4* [23]. In 2015, Guo et al. showed that the expression of *OsPHR3* gene was induced under Pi starvation, but not that of *OsPHR1/2* [22]. Additionally, all three OsPHRs exhibit different DNA-binding affinity properties, and only plants with overexpression of *OsPHR3* gene exhibited low-Pi stress tolerance under field conditions. They proved that functional redundancy exists between OsPHR1, OsPHR2, and OsPHR3 proteins and such diversity enables them to co-regulate Pi response in rice [22]. Further, it was shown that similar to *OsPHR3, OsPHR4* is a Pi starvation-induced gene and its expression is directly regulated by OsPHR1/2/3, which can all bind to the P1BS elements located in the *OsPHR4* promoter [23]. Interestingly, OsPHR4 could also bind to its own promoter in this study.

### 2.2. PHRs Work Together in a Link

Beside PHR redundancy, dimerization itself is a crucial step for PHR-like TF DNA binding capability. Previous reports showed that AtPHR1 forms heterodimers with AtPHL1 [11], and the interaction of AtPHL2 and AtPHL3 was also observed, and both can homodimerize [18]. Likewise, Ruan et al. showed that OsPHR4 could form a heterodimer with either OsPHR1, OsPHR2, or OsPHR3, as well as homodimers [23]. The nuclear-localized homodimerization of OsPHR2 protein was also reported [7,24]. All these findings suggest that PHR-like TFs can act redundantly and form an integrated system in Pi-starvation signaling in plants.

## 3. The Multifunctional Role of PHR1

The main idea of this review is to point out recent findings around PHR protein family members except their self-evident DNA-binding role. However, it is worth to mention that PHR transcription factors target broad range of genes that are not connected directly with the Pi signaling. Here, we would like to present relevant studies concerning PHR1 role in various biological processes in plants.

### 3.1. PHR1 Affects Plant Immune System

P deficiency makes plants more sensitive and susceptible to become a host of various phytopathogens [25,26]. Thus, the plant immunity system has to react immediately to overcome the severe environmental stimuli through changing the composition of hormones and root exudates. PHR1 TF as a major regulator of *PSI* genes also affects the expression of genes involved in antimicrobial resistance. Antagonistic interactions between three plant hormones: (i) salicylic acid (SA), (ii) jasmonic acid (JA), and (iii) ethylene (ET) trigger resistance against pathogens and herbivory [27,28]. JA induction shares some typical traits observed in Pi-starved plants, such as: anthocyanin accumulation or growth reduction [29], suggesting that both signaling pathways may be connected [30].

The comprehensive data about the contribution of PHR1 to the transcriptional regulation of plant immunity-related (PIR) genes has been published in last few years. In 2016, Khan et al. showed the significant increase of JA level in *Arabidopsis* leaves and roots under low-Pi treatment [31]. Additionally, the molecular analysis of loss-of-function *phr1-1* mutant revealed that this induction may be partially controlled by the PHR1 TF. The activation of JA signaling pathway upon Pi deficiency was delayed in the *phr1-1* mutant, but not abolished completely, indicating the presence of other transcription factors that may regulate this process [31]. One year later, global ChIP-seq (chromatin immuno-precipitation-sequencing) experiment published by Castrillo et al. uncovered the significant enrichment in clusters of JA- and SA-related genes involved in plant defense, targeted by *PHR1* in *Arabidopsis* [32]. In *phr1* and *phr1 phl1* mutants most of the SA-responsive genes were upregulated compared to wild type. Where for majority of JA-responsive genes their expression was lower in *Arabidopsis* mutants than in wild type. Further, they found that *phr1 phl1* double mutants exhibit enhanced activation of plant immunity, suggesting the repressing role of AtPHR1/AtPHL1 TFs on plant immune system [32]. These results are consist with related reports showing, (i) that transcription of ET biosynthesis genes may be affected by AtPHR1 activity [11] and (ii) a group of candidate genes involved in SA, JA, and ET signal transduction were differentially expressed upon Pi deficiency in sorghum [33].

Moreover, PHR1-dependent phosphate starvation responses (PSR) may be altered by root microbial communities in *Arabidopsis*. It was shown that intact PSR suppress the root colonization by fungal root endophytes [34], where synthetic bacterial community (SynCom) triggers PHR1 activity in low-Pi conditions [32].

### 3.2. Metal-Phosphate Relationship Modulated by PHR1

In soils, metal cations (i.e., Ca^2+^, Zn^2+^, Fe^3+^) form insoluble precipitates with the inorganic forms of phosphate impeding the availability of these elements for plants [35,36,37]. While in plant cells, metal homeostasis involves interactions with enzymes and organic macromolecules as well as negatively charged Pi altering its activity. Extracellular Pi level also can affect the concentration of metal ions acquired by plant root system. For example, the expression of gene encoding highly conserved ferretin 1 (FER1) iron-binding protein is strongly induced upon Pi scarcity [38]. Bournier et al. found that *Arabidopsis phr1 phl1* loss-of-function mutant accumulates iron upon Pi deficiency [39]. Interestingly, the low-Pi induced expression of *AtFER1* gene was completely lost and different cellular patterns of iron distribution were observed. They showed that both PHR1 and PHL1 proteins directly bind to the P1BS motif within the promoter of *AtFER1* gene inducing its transcription under Pi deficiency, in a Pi-specific manner [39]. Cross-talk between Pi and zinc signaling has been also recognized in the PHR1-dependent manner. Usually, P and Zn elements are present in a small amount in the soils and are barely available for plants. The transcription of two genes encoding zinc transporters (*ZIP2* and *ZIP4*) is positively regulated by PHR1 TF [40]. Besides, comparative analysis of the collections of transcriptomic data highlighted the PHR1-dependent induction of candidate genes involved in calcium signaling in Pi-depleted roots as well [41].

### 3.3. Double-Faced Role of PHR1 in the Regulation of Sulfate Homeostasis

In addition, few reports also describe the involvement of PHR1 TF into the transcription of non-metal ions homeostasis. Rouached et al. showed the involvement of PHR1 TF into the sulfate transfer from shoot to root during Pi starvation [16]. They found the presence of P1BS motifs within two genes (*SULTR1;3* and *SULTR2;1*) encoding sulfate transporters. Further, molecular analysis of *phr1* mutant revealed that PHR1 TF plays both a positive and negative role on the expression of genes encoding sulfate transporters. They observed the induction of the *SULTR1;3* gene expression upon low-Pi, but repression of the *SULTR2;1* and *SULTR3;4* (not P1BS holder) genes expression in *Arabidopsis* [16,42].

## 4. Transcriptional Regulation of *PHR1* Gene Expression

Broadly, signaling pathways recruit TFs, which function as a last executor in the stepwise action leading to precise changes in target gene expression. However, long distance or systemic sensing pathways trigger each other and recruit a wide range of TFs to coordinately manage the steady state of living cells. In *Arabidopsis* and barley, *PHR1* gene expression is not particularly Pi responsive and its transcript level was not seen to change in different Pi regimes [6,43].

### PHR1 Promoter as a Station for Many Plant TFs

**Figure 1:**
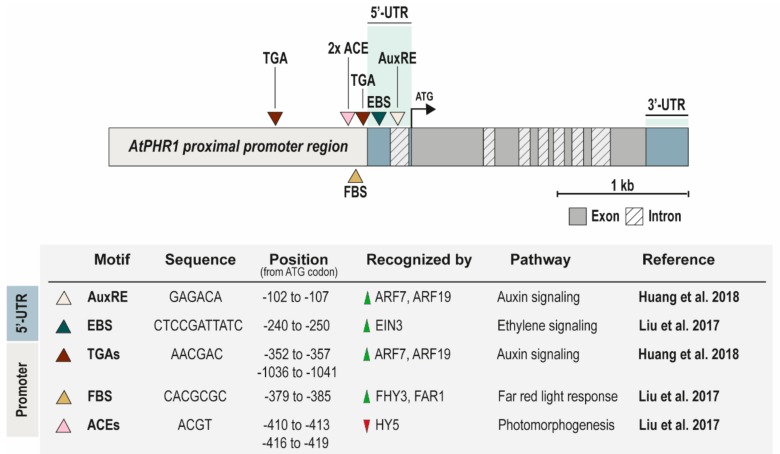
*Arabidopsis PHR1* gene structure with outlined major *cis*-regulatory motifs. Summary table provides detailed information about all relevant motifs published recently. Green and red triangles in column: “recognized by”, depict up- and down-regulation of *PHR1* genes expression by particular transcription factors (TFs), respectively.

Recently, several TFs that regulate *AtPHR1* gene expression in various conditions have been uncovered. In 2017, Liu et al. identified a few *cis*-regulatory elements within the *AtPHR1* promoter, including two elongated hypocotyl 5 (HY5 TF) binding sites (ACGT-containing elements (ACEs) [44]), one far-red elongated hypocotyl 3 (FHY3 TF), and far-red-impaired response 1 (FAR1 TF) binding site (FBS; CACGCGC [45]), and a palindromic repeat sequence similar to the ethylene-insensitive 3 (EIN3 TF) binding site (EBS) [46]) [47]. They observed that *AtPHR1* gene expression is induced by light. Additionally, *AtPHR1* transcript levels were positively correlated with the intensity of light, and the expression levels of eight PSI target genes, activated by PHR1, were significantly lower in dark-grown plants compared with light-grown (Figure 1). Extensive work by Liu’s group proved that FHY3 and FAR1 TFs positively regulate and HY5 TF negatively regulates *AtPHR1* expression and PSI genes [47]. What is more, another analysis showed that the transcript levels of *AtPHR1* and the PSI genes were enhanced by 1-aminocyclopropane-1-carboxylate (ACC; the immediate precursor of ethylene) treatment and Pi deficiency enhances plant sensitivity to ethylene, as reflected by induction of PSI gene expression [47,48]. Such cross-talk is very likely mediated by EIN3 TF, which directly binds to the *AtPHR1* 5′-UTR and specifically recognize the EBS sequence. The FHY3 and EIN3 TFs form a complex and together coordinately regulate *AtPHR1* expression in response to both light and ethylene stimulus [47]. Recently, many groups have shown that low-Pi induced responses integrate ethylene signaling into the molecular network, which helps to remodel the root architecture and increase Pi mining capability [47,49,50,51]. Later, Huang et al. identified three auxin-response elements: one copy of the AuxRE (GAGACA) in 5′-UTR and two copies of the TGA (AACGAC) elements in the promoter (Figure 1). They found also that two auxin response factors, ARF7 and ARF19, bind to these DNA motifs within *AtPHR1* 5′-UTR and promoter sequences to positively regulate its gene expression. Further, the ARF7/ARF19 expression patterns in roots are similar to that of *AtPHR1* gene [52]. Interestingly, Huang’s group found auxin-response elements in the promoters of most MYB-CC family genes in *Arabidopsis*, which were confirmed to exhibit functional redundancy to AtPHR1 protein [18,19,52]. They proposed a model in which plants exposed to Pi scarcity showed increased sensitivity of an auxin receptor, transport inhibitor response 1 (TIR1), which led to upregulation of ARF7/ARF19 TFs following the induction of *AtPHR1* expression and their *PSI* target genes in roots [52]. Year by year we get clues suggesting that in order to understand the role of PHR-like TFs in maintaining phosphate homeostasis, we need to look extensively at every single step of the signal transduction pathway.

## 5. PHR1 Post-Translational Modifications

Post-translational modification (PTM) is a biochemical modification that occurs to one or more amino acids on a translated protein. Such modification is mostly catalyzed by enzymes that recognize specific target sequences, and may determine the secondary structure of the target proteins and their subcellular localization, activity, and stability [53]. One of the most common and evolutionarily conserved PTMs in eukaryotic cells is mono- or poly-SUMOylation, which involves the binding of small ubiquitin-related modifier (SUMO) protein. The SUMO protein, with an average 10 kDa molecular mass, leads to increased target protein mass or spatial surface related to protein–protein and protein–DNA interactions [54]. Previous work established that PHR-like TFs are SUMOylated via SIZ1 (SAP and MIZ/SP-RING zinc finger domain-containing protein 1) SUMO E3 ligase in *Arabidopsis* [55], rice [56], and *Malus domestica* (Borkh.) [57]. The pioneering work of Miura’s group confirmed that *AtSIZ1* is a single-gene family that encodes protein localized to nuclear speckles in *Arabidopsis* cells. The *siz1* loss-of-function mutant exhibits symptoms that are associated with Pi deficiency, such as reduced primary root growth and increased lateral root and root hair length and density, higher root/shoot mass ratio, anthocyanin accumulation, and upregulation of PSI gene expression [55]. There are two lysine residues within the AtPHR1 amino acid sequence, in positions 261 and 372, that are crucial for SUMO binding, and it was proved that K261R and K372R mutations prevent SUMOylation of PHR1 [55].

The expression level of *AtPHR1* gene is relatively stable during Pi deficiency. So far, many groups have suggested that AtSIZ1-conducted SUMOylation stabilizes the level and activity of AtPHR1 protein and accelerates its binding affinity to the P1BS motifs present in the regulatory regions of PSI target genes, such as *AtIPS1* (*INDUCED BY PHOSPHATE STARVATION 1*) and *AtRNS1* (*RIBONUCLEASE 1*) [55,56,57]. However, mutation of *OsSIZ1* gene revealed a dual role of SIZ1 E3 ligase in the regulation of Pi homeostasis in rice. Among 13 high-affinity Pi transporters (PHT1 protein family) in rice, *OsPT1* and *OsPT8* gene expression was induced in *siz1* rice mutants under Pi deficiency. On the contrary, suppression of *PSI* genes such as *OsPT2* and *OsPT6* was also observed in this study [56]. Thus, AtSIZ1 and OsSIZ1 can act negatively or positively on the expression of *PSI* genes, even on genes that are not targeted by PHR1 transcription factors. Because of the lack of data, we can only speculate that various SIZ1-SUMOylated transcription factors work together in response to diverse environmental stresses in plants [55,58,59,60,61,62]. So far, there are no data on other PTMs that may affect PHR1 activity.

## 6. PHR1 Meets Nitrogen and Phosphate Sensors

**Figure 2:**
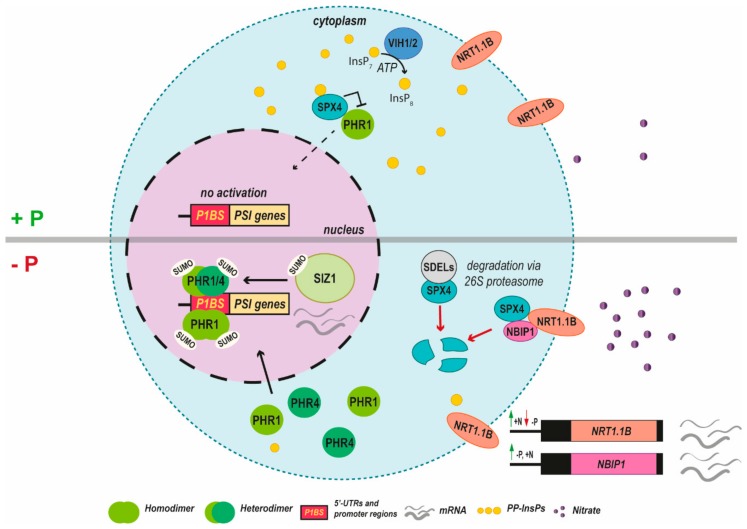
Graphical overview of interplays between PHR-like TFs and other cellular components under Pi scarcity in plant cells updated with current knowledge obtained from extensive research in *Arabidopsis* and rice. PHR1 TF represents both AtPHR1 and its rice orthologue OsPHR2. Under sufficient phosphate levels (+P), various inositol pyrophosphate isomers (PP-InsPs, depicted as yellow dots of different sizes) are biosynthesized to mimic the cellular Pi status. In the presence of Pi and ATP, InsP_8_ messenger molecules are generated by the activity of kinase domain within VIH1/2 enzymes. Such PP-InsPs isomers (InsP_6_, InsP_7_, InsP_8_) compete to bind to the SPX-domain containing proteins, followed by direct inhibition of AtPHR1/OsPHR2 nucleo-cytoplasmic shuttling. This leads to no activation of PSI genes. When the Pi level turns down, the protein level of specific E3 ligases, such as SPX4 degradation E3 ligases 1 or 2 (SDEL1, SDEL2), increases to target SPX-domain containing proteins for proteasomal degradation pathway. *NRT1.1B* is transcriptionally induced by high nitrate (N) or repressed by low-Pi, where *NBIP1* gene is upregulated by both low-Pi and high-N. The nitrate transporter 1.1B (NRT1.1B) trans-membrane nitrate sensor mediates nitrate-triggered SPX4 degradation with NBIP1 E3 ligase in phosphate signaling upon N sufficient conditions. Upon low-Pi, the PHR-like TFs are more preferentially localized in the nucleus and their structure is stabilized by SUMOylation conducted via SIZ1 activity. The PHR-like TFs, as either homo- or heterodimers, can regulate the transcript level of PSI genes by binding to the P1BS motifs present in the gene 5′-UTR or promoter regions. Green and red arrows indicate up- and down-regulation of gene expression, respectively.

The concentration of nutrients in the plant tissues is determined by nutrient-specific overlapping pathways that cooperate to balance nitrogen (N) and P uptake [63,64,65]. Fertilizers with additive N macroelement can increase the plants’ P uptake and the proper N:P supply ratio, making it essential for promoting plant growth and subsequent high crop yields [66,67]. Variations in nutrient availability can alter specific gene expression levels or even activate the expression of genes that were inactive before. Related studies on OsPHR3 TF revealed that it is responsive to different forms of N irrespective of Pi regime. Sun’s group raised the hypothesis that *OsPHR1/2/3/4* genes can also take part in the cross-talk between N and P [42]. In this part, we would like to point out major research breakthroughs that were made in last few years and connect PHR-mediated phosphate responses with nitrate signaling (Figure 2).

### 6.1. SPX Proteins Navigate PHR1 in Plant Cells

Another way the Pi-related regulation of PHR1 transcriptional activity exists is through interaction with proteins containing SPX (a name combining suppressor of yeast GPA1 (SYG1), CDK inhibitor in yeast PHO pathway (Pho81), and xenotropic and polytropic retrovirus receptor (XPR1)) domains [8,68,69,70]. There are four SPX proteins in *Arabidopsis*, AtSPX1–AtSPX4 [71,72,73], and six in rice, OsSPX1–OsSPX6 [8,68,74,75]. The *AtSPX* genes are highly homologous, however various expression patterns and subcellular localizations were described for them, indicating their functional diversity [70]. A nuclear protein AtSPX1 sequesters AtPHR1 in a Pi-dependent manner and inhibits its activity in *Arabidopsis*. In Pi-starved plants, AtSPX1/2/3 proteins are quickly degraded by the 26S proteasome pathway and AtPHR1 can freely regulate the expression of PSI genes. While the increasing Pi levels enhance the AtSPX1 protein half-life, they could preferentially interact with AtPHR1 TF, diminishing AtPHR1 binding capability to the P1BS *cis*-elements. Interestingly, SPX1–SPX3 possess P1BS motifs within their 5′-UTR and/or promoter region and serve as downstream targets of AtPHR1/OsPHR2 proteins. Thus, PHR1 can guide its own central role in Pi sensing by this negative feedback loop [70,71,76,77].

In rice, recent findings proved that contrary to nuclear AtSPX1/OsSPX1 proteins, OsSPX4 localizes in both the cell nucleus and cytoplasm, and it is not Pi-starvation responsive [24]. OsSPX4 physically interacts with OsPHR2 mainly in the cytoplasm, where such action prevents the nucleo-cytoplasmic shutting of OsPHR2 in the presence of Pi. Afterwards, when OsPHR2 is trapped in the cytoplasm, it cannot form homodimers and binds to P1BS motifs, and in consequence the PHR-mediated signal transduction is stopped (Figure 2) [24].

### 6.2. Inositol Pyrophosphates (PP-InsPs) as Messenger

Throughout the paper we have shown many examples of how changes in the Pi level can affect a particular gene’s expression and intracellular responses and turn the mode of action in plant development and adaptation to environmental stimuli. It raises the hypothesis that there should be a kind of universal signal element that can sense the signal transduction in response to Pi availability. Among the well-known secondary messengers for a variety of stimuli in eukaryotic cells (also common in plants) are cytosolic calcium ions, Ca^2+^. In the calcium signal transduction pathway, four Ca^2+^ ions target and activate calcium-binding messenger protein, calmodulin (CaM), which modulates subsequent protein–protein interactions [78,79,80]. Calcium ions were mentioned for a reason, because specific inositol 1,4,5-triyphosphates (InsP_3_) bind to the ligand-gated calcium channels and trigger the release of stored Ca^2+^ ions [81,82]. InsP_3_ can be further phosphorylated to InsP_4_ [83], InsP_5_, InsP_6_ [84,85,86], InsP_7_ [87], and InsP_8_ [88].

According to related research reports, especially the inositol pyrophosphates on the highest level of phosphorylation (PP-InsPs) play an important role coordinating cellular Pi homeostasis in plants [82,86,87,88]. Dong et al. showed that InsP_8_ directly binds to the SPX domain and regulates the interaction between SPX1 and PHR1 in *Arabidopsis*. They demonstrated that in mutant plants exhibiting no ability to biosynthesize InsP_8_, the SPX1–PHR1 complex could not be formed, which resulted in the constitutive activation of PSI genes and overaccumulation of Pi [88]. Recently, biochemical studies have revealed various binding affinities between InsP_6_ and InsP_7_ to the SPX domain and competition of PP-InsP isomers prevailing over the physiological concentration of Pi. The elaborated crystal structure of SPX domain exposed some features of binding surface targeted by PP-InsPs. InsP_6_ interacts with the SPX domain via variable hydrogen bond interactions, which may sense different PP-InsP isomers [87]. As far as *PHR1* gene expression is not regulated by Pi status, its activity can be fine-tuned by the presence of specific SPX-InsP complexes in an intracellular Pi-dependent manner.

The bioenergetics and signaling roles of PP-InsP molecules are evolutionarily more ancient than InsP_3_-mediated Ca^2+^ mobilization [89]. Plants exposed to low-Pi stress reprogram their metabolic pathways to compensate for cellular energetic crisis through the coordination of 5-InsP_7_ or InsP_8_ levels. In 2019, Zhu et al. reported that two genes encoding inositol pyrophosphate kinases/phosphatases VIP homolog 1/2 (*VIH1/2*) were able to either generate or break down PP-InsPs in *Arabidopsis* [90]. The VIH1/2 enzymes are bifunctional, harboring an N-terminal InsP kinase and a C-terminal phosphatase domain [91]. The point mutation within the active site of the kinase domain leads to overaccumulation of Pi and constitutive Pi starvation responses. Further phosphorylated PP-InsP isomers cannot be catalyzed, and they do not mediate SPX4-PHR1/PHL1 arrest. Deletion of either *PHR1* or *PHL1* can partially rescue the *VIH1-2 VIH2-4* double-mutant phenotype, suggesting that both enzymes redundantly regulate Pi homeostasis and their PP-InsP reaction products are part of the PHR1/PHL1 signaling cascade. The dual roles of VIH1/2 may be shaped by cellular concentration of ATP and Pi [90]. For example, plants growing in soil with sufficient Pi availability are energetically stable, and their increasing cellular level of ATP stimulates PP-InsP kinase activity. Thus, InsP_8_ isomers are more abundant and act as messengers that transmit information about Pi availability throughout the plant, so PSI responses stay inactive.

Plant hormones may interplay with Pi signaling also through interactions with different InsP isomers. Recent discoveries have shown the possibility of binding either InsP_5_ to the JA receptors [92] or InsP_6_ to the auxin receptors [93]. Furthermore, herbivore-induced JA synthesis triggers VIH2-dependent increase in InsP_8_, which can be integrated into JA receptor complex [94].

### 6.3. SPX Proteins from the Nitrogen Perspective

Recently, two breakthrough studies appeared that extended the SPX4-PHR2 module for novel molecules that were known as nitrate sensors. Maeda’s and Hu’s groups revealed the mechanism by which nitrate activates both Pi and N signaling pathways in plants [75,95]. Maeda et al. found three copies of P1BS *cis*-regulatory elements in the nitrate-inducible GARP-type transcriptional repressor 1.1 (*AtNIGT1.1*) promoter, which encodes nuclear localized TF transcriptionally regulated by nitrate. Further investigation of the SPX-PHR1-NIGT1 cascade revealed their role in the modulation of nitrate uptake in a P-dependent manner. Additionally, they proved that the transmembrane protein and nitrate sensor, the nitrate transporter 1.1B (NRT1.1B), recruits SPX4 protein to facilitate its ubiquitination and degradation mediated by NRT1.1B interacting protein 1 (NBIP1) E3 ligase in the presence of N in rice (Figure 2) [75]. In *Arabidopsis*, NRT1.1 activity is positively and indirectly regulated by the phosphate 2 (PHO2) ubiquitin-conjugating (UBC) E2 enzyme [96,97]. In a short period of time, two more SPX4 degradation E3 ligases (SDEL1 and SDEL2) were discovered. Both *SDEL* genes are post-transcriptionally induced by Pi starvation, and their E3 ligase activity directs for degradation SPX4 proteins via ubiquitination of K213 and K299 lysine residues (Figure 2) [69]. Again, it was proved that PHR-like TF functions as a master regulator to maintain nutrient homeostasis in plants. The AtPHR1/OsPHR2 TFs could compete with various E3 ligases by interacting with SPX4-PP-InsP-activated protein in either a P- or N-dependent manner, which secures SPX4 from being directed to the 26S proteasomal degradation pathway [69,75]. The plant demand for P strengthens through developmental stages in which large macromolecules, nucleic acids, and proteins are created from smaller components extensively. Inhibition of N uptake can lower the plant’s demand for P and cooperatively reduce the negative impact of abiotic stresses.

## 7. Conclusions

Thus far, the given findings indicate that PHR1 transcription factor is a crucial component of Pi signaling in plants. Here, we emphasize the role of cooperation between signaling and hormonal pathways that are most affected by Pi-starved plants. Alterations by addition or deletion of any factor(s) from a signal transduction cascade can result in sudden cellular and molecular changes. We reviewed several breakthrough studies that should be considered during further investigation of the mechanistic picture determining plant tolerance to phosphate scarcity from the perspective of the PHR protein family. The first described PHR protein family member, PHR1 in *Arabidopsis*, redundantly cooperates with other homologous proteins (i.e., AtPHR2, AtPHL1, AtPHL2), which may equally contribute to the signaling pathway. It is believed that specific dimer sets consisting of PHR-like TFs may regulate the expression of essential genes, overcoming the negative impact of low-Pi stress.

The “phosphate problem” has recently gained much attention due to the anthropogenic impact on the environment and limited phosphorus supply. Crop improvement in current plant breeding will occur due to revealing the mechanism of Pi tolerance. Such knowledge may be used to engineer crop cultivars with improved ability to acquire and utilize Pi. In the near future, efforts should be put into investigating the relationship between different PP-InsP isomers that may coordinate cellular phosphate balance with metabolic messengers. These evolutionarily conserved signaling molecules speak directly on behalf of plants, and decoding this language may be invaluable.
